# Design of Wideband FMCW Radar Transceiver System Using Photonic Elements

**DOI:** 10.3390/mi14071296

**Published:** 2023-06-24

**Authors:** Sungjun Yoo, Youngseok Bae, Sunghoon Jang

**Affiliations:** Agency for Defense Development, Yuseong, P.O. Box 35, Daejeon 34186, Republic of Korea

**Keywords:** microwave photonics, radar transceiver system, photonic based radar

## Abstract

This paper proposes a FMCW radar transceiver with photonic elements. The proposed radar system is efficiently designed by budget analysis, and a wideband signal is generated using photonic elements. To verify the performance of the proposed radar system, field tests including changes in bandwidth are conducted. The results confirm that the resolution of ISAR images improves as the bandwidth increases as expected through the budget analysis.

## 1. Introduction

As the integration of low-probability of intercept (LPI) technology into unmanned aerial vehicle (UAV) gradually increases with the development of relevant technologies [1,2,3], the demand for research to prevent the threats posed for these aircraft is growing [4], and to detect the target in advance is of paramount importance. However, the detection of LPI targets, which are small and have low radar cross-sections (RCS), is difficult when using conventional radar systems. A radar transceiver system using photonics technology has recently attracted attention as a fundamental solution to the LPI target detection issue by using the capability of wideband signal generation [5]. Microwave photonic radar technology has some advantages in low-frequency-dependent loss and wideband frequency characteristics, and this technology has been adopted for obtaining high-frequency signals for signal processing using photonics elements. In [6], a photonic-based phased array radar architecture is proposed to obtain the target image through digital beamforming. Moreover, various studies have been conducted including increase in detection range [7,8], use of high-frequency bands for low-RCS target detection [9,10,11]. In addition, there have been substantial efforts to implement various systems using microwave photonics technology for high-resolution images [12,13,14,15]. For example, in [14], a linearly chirped microwave generator using photonic elements was introduced for SAR systems with large-range resolution demand, such as Earth surveillance and monitoring. In [15], the use of photonic generation and transmission of linearly chirped microwave waveform was examined for modern radar systems.

However, most of the preceding studies have remained at the level of showing the feasibility of microwave photonics by employing indoor experiments [9,10,11,12,13]. Although several researchers have conducted experiments to verify the performance of the technology in a real environment, most of their studies have used targets with large RCS such as airplanes [8]. Thus, these results are not suitable for LPI targets with low RCS, and such research has limitations in detecting and identifying a target in a real-field environment.

In this paper, we propose a frequency modulated continuous wave (FMCW) radar transceiver using photonic elements with wideband property to obtain high-resolution images. The proposed transceiver system with photonic elements does not use various electrical elements such as mixers and filters which are used in conventional radar transceiver systems to obtain wideband characteristics with low-frequency-dependent loss [15]. To immediately apply the proposed system to a real environment, the performance of the transceiver system is observed and optimized using budget analysis and simulators. In order to verify the proposed system in terms of detection and inverse synthetic aperture radar (ISAR) images acquisition, the proposed system is fabricated and its performance is verified in real-field test conditions. The results demonstrate that the proposed radar transceiver with photonic elements is suitable for radar systems aiming to detect low-RCS targets and high-resolution images.

## 2. Proposed Radar System Design

**Figure 1:**
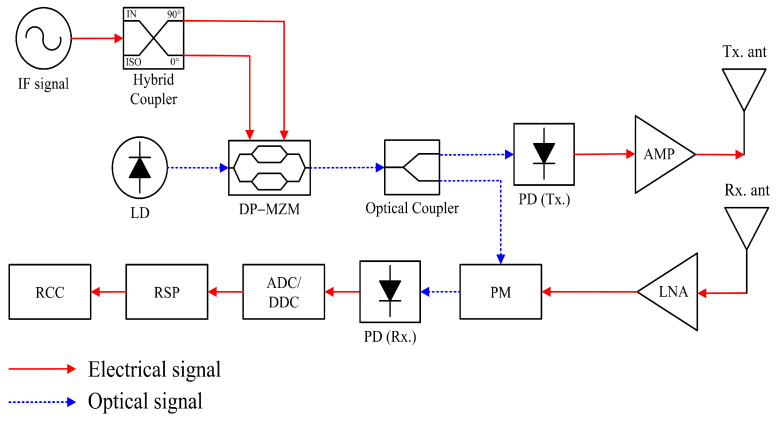
Schematic of the microwave photonic FMCW radar transceiver.

Figure 1 shows the schematic of the proposed microwave photonic FMCW radar transceiver with photonic elements for obtaining wideband characteristics. The proposed radar transceiver consists of a laser diode (LD), RF signal, dual-parallel Mach–Zehnder modulator (DP-MZM), optical coupler, phase modulator (PM), photo detector (PD), transmit and receive antennas, amplifier (AMP), low noise amplifier (LNA), analog-to-digital converter (ADC), radar signal processing (RSP), and a radar control center (RCC). The photonic elements, such as LD, DP-MZM, and PM are implemented in the proposed FMCW radar transceiver to achieve wideband properties, and these allow us to obtain high-resolution ISAR images.

A continuous wave light from the LD (model: AA1401, Gooch & Housego, Dowlish Ford, Limister, UK) is modulated by the DP-MZM, which is driven by a continuous wave (CW) linear frequency modulation (LFM) signal in the intermediate frequency band (IF-band). The frequency of the LFM signal in the IF-band can be expressed as:

(1) $$f_{IF}\left(t\right)=f_{0}+kt$$

where *f*_0_ is the initial frequency and *k* is the chirp rate. The IF signal passes through an electrical 90° hybrid coupler, and the two signals with 90° phase difference are sent to drive the DP-MZM. Bias voltages of the DP-MZM are properly set to obtain second-order modulation, and the output optical signal of the DP-MZM is then split into two branches by an optical coupler.

In the transmission path, the optical signal is converted to an electrical signal using a PD, and the obtained electrical signal has a quadrupled LFM signal compared with that of the LFM signal in the IF-band. The frequency of the quadrupled LFM signal can be expressed as follows [9]:

(2) $$f_{LFM}\left(t\right)=4f_{0}+4kt$$

In the receiving path, the optical signal split by the optical coupler from transmission is used as a reference for the de-chirping process to obtain beat frequency for the FMCW radar transceiver system. The beat signal is obtained by combining the transmitted signal and backscattered signal in the receiving path, and the calculated signal can be used for target detection and ISAR image acquisition by using digital signal processing (DSP). The proposed transceiver system with photonic elements does not use various electrical elements such as mixers and filters that are used in conventional radar transceiver systems, and consequently it has some advantages such as low-frequency-dependent loss, wide bandwidth, tunability for operating frequency, and immunity to electromagnetic interference (EMI) [5].

We also observe the optical and the electrical signal of the proposed radar transceiver system using a photonic multiphysics simulation tool (Ansys Lumerical, Vancouver, BC, Canada) to demonstrate the effectiveness of achieving wideband characteristics using photonic elements.

**Figure 2:**
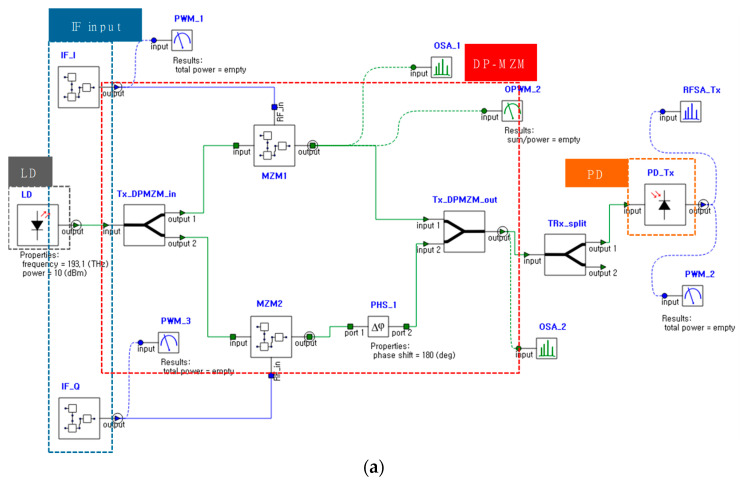

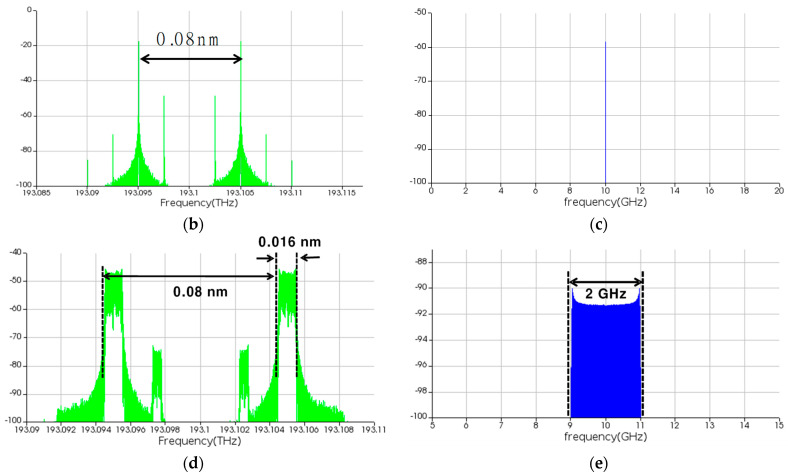
(**a**) Simulation circuit by Lumerical Interconnect. (**b**) Simulation output result from OSA with an input ISF CW signal of 2.5 GHz. (**c**) Simulation output result from ESA with an input IF CW signal of 2.5 GHz. (**d**) Simulation output result from OSA with an input IF LFM signal of 2.5 GHz. (**e**) Simulation output result from ESA with an input IF LFM signal of 2.5 GHz center frequency and 500 MHz bandwidth.

Figure 2a shows the simulation circuit of the proposed radar transceiver. The laser wavelength is 1550 nm with an optical power of 10 dBm and a linewidth of 100 kHz, and the bias voltage points of the DP-MZM are adjusted to observe the optical power. The property of the PD assumes that responsivity and dark current are 1 A/W and 0.01 A, respectively. The simulation results are measured in the optical domain (before PD) and electrical domain (after PD), respectively. Figure 2b,c show the simulation results from the optical spectrum analyzer (OSA) and the electrical spectrum analyzer (ESA), respectively, when an input IF CW signal has a frequency of 2.5 GHz. To obtain the second-order modulation of DP-MZM, the three bias voltages should be controlled, and the sub-MZMs are biased at the full point, and the outer parent of MZM is biased at the null point with a phase shifter when the optical carrier is equally divided into two parallel children sun-MZMs. As can be expected from Equation (2), the distance between the two second-order sidebands is 0.08 nm, which allows the proposed system to generate a 10 GHz signal [9].

The signal generation simulation has been also conducted for the LFM signal which will be applied in the proposed system. Figure 2d,e present the simulation results from the OSA and the ESA, respectively, when the input IF LFM signal has 500 MHz bandwidth at 2.5 GHz center frequency. The results demonstrate that the proposed transceiver can generate a quadrupled signal while the fundamental optical carrier and two first-order sidebands are suppressed.

To detect low-RCS targets and obtain high-resolution images when RCS is below 1 m^2^, the link budget of the proposed transceiver was calculated when considering the characteristics of the RF electronics and the photonic elements. The parameters and their values are specified in Table 1.

**Figure 3:**
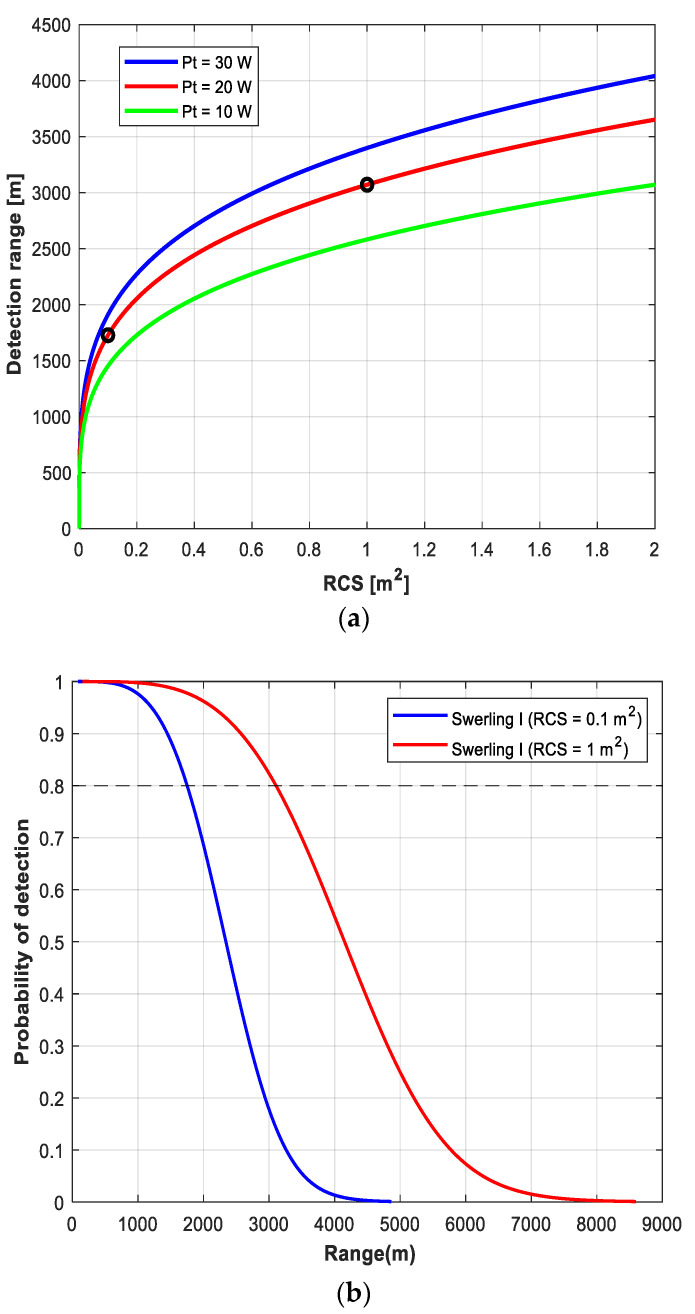
(**a**) Detection range versus RCS plot with changes in transmission power. (**b**) Detection probability versus range plot with changes in RCS.

The maximum detection range of a target depends on the RCS of the target and the peak power of the radar. The maximum detection range can be arranged as

(3) $$R_{max}={\left(\frac{P_{t}G_{t}G_{r}G_{p}\lambda^{2}\sigma }{{\left(4\pi \right)}^{3}KTBFL_{T}\left(\min\left(S/R\right)\right)}\right)}^{\frac{1}{4}}$$

where *σ* and *B* refer to the RCS of the target and the instantaneous receiver bandwidth, respectively. The other variables are as described in Table 1. Figure 3a presents maximum detection ranges according to RCS, and the ranges are expected to be 3000 m and 1800 m when RCS is 1 m^2^ and 0.1 m^2^, respectively, with *P_t_* of 20 W, as indicated by the circles (‘o’).

The RCS of a target changes every moment according to the movement of the target relative to the radar, resulting in the fluctuation loss. The Swerling model addresses this issue by describing the relationship of the target RCS and the probability density function (PDF) using the chi-square distribution. The Swerling I model was used in this study, and the PDF in this case can be expressed as

(4) $$P_{d}=\exp\left(-V_{T}/\left(1+S/R\right)\right)$$

where *P_d_*, *V_T_*, and *S*/*R* refer to the probability of detection, detection threshold, and signal to noise ratio, respectively [16]. Here, we assumed the number of integrated pulses is 1. Figure 3b illustrates the detection probability for moving targets when *P_d_* is 0.8 with RCS of 0.1 m^2^ and 1 m^2^ [17].

## 3. Measurement and Analysis

**Figure 4:**
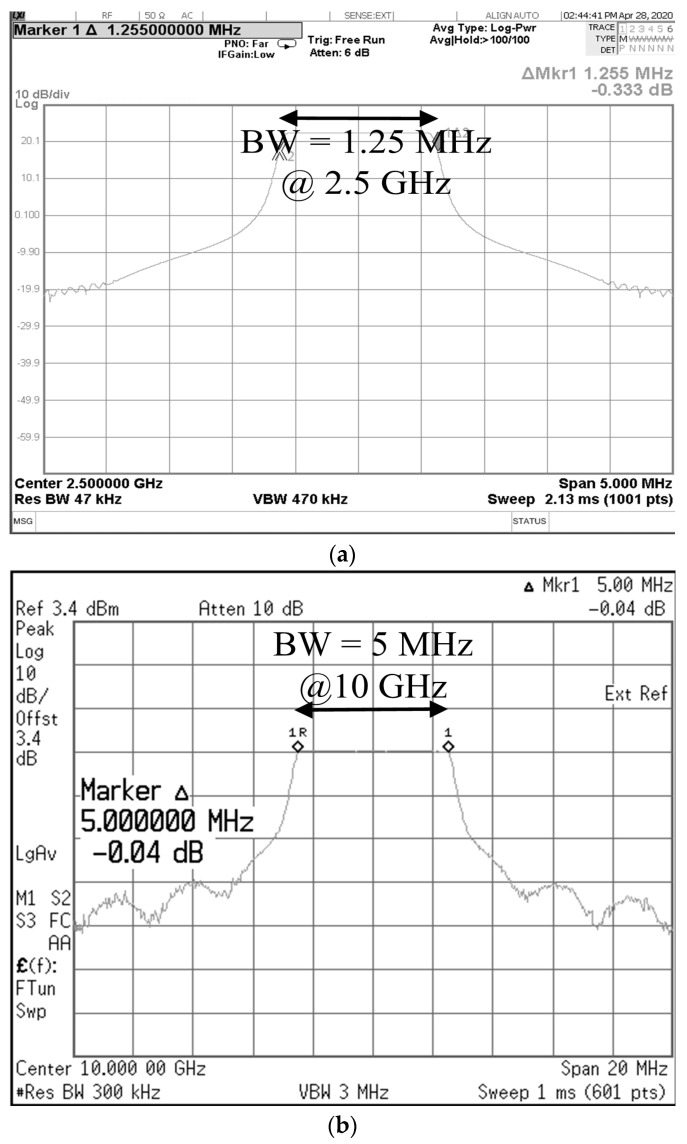
Measured center frequency and bandwidth of the (**a**) IF signal and (**b**) the quadrupled transmit signal applied to the antenna.

Figure 4 shows the signal generated in the transmission path; that is, the input signal to Tx. The center frequency and bandwidth of the initial IF signal are 2.5 GHz and 1.25 MHz, respectively. The transmit signal applied to the antennas is centered at 10 GHz with a bandwidth of 5 MHz that is quadruple the value of that of the IF signal, and the result demonstrates that the proposed transceiver can achieve wideband characteristics using photonic elements.

Moreover, the measured full width at half maximum (FWHM) are 1.27 MHz at 2.5 GHz and 7 MHz at 10 GHz, respectively, which verify that the generated signal is designed close to the rectangular waveform. The bandwidth of 5 MHz is used in the target detection mode in the proposed radar transceiver system, and the range resolution is 2.4 m when the pulse repetition interval is 200 μs, and the number of the range bin is 4096 [16].

**Figure 5:**
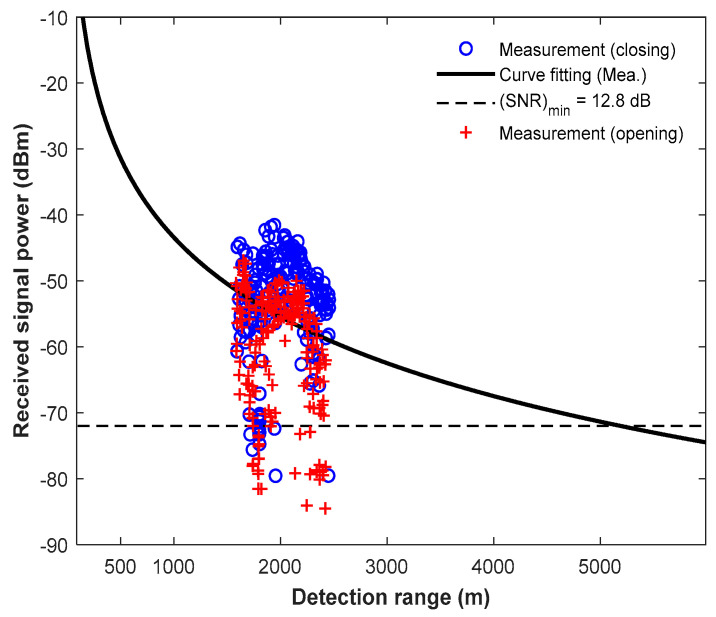
Received signal powers of closing and opening according to the detection range with curve fitting assuming a target RCS of 0.1 m^2^.

Figure 5 presents received signal powers according to the detection range, with the measured closing (approaching the radar system) and opening (away from the radar system) results specified as ‘o’ and ‘+’, respectively. The closing signal power is bigger than the opening signal power, and the curve fitting assuming the target’s RCS is 0.1 m^2^ is represented as the solid line. In the experiment, the minimum SNR is estimated to be 12.8 dB, which is in good agreement with the simulation result of 13 dB. In addition, the maximum target range is estimated to be more than 5 km. Therefore, these results demonstrate that the requirements of the maximum detection range are satisfied as listed in Table 1.

**Figure 6:**
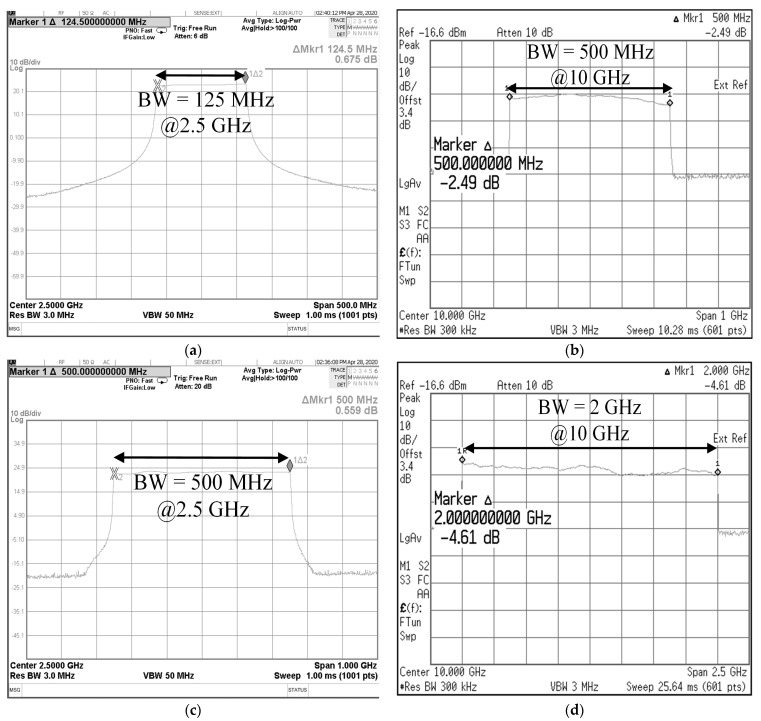
Measured (**a**) IF signal with 125 MHz bandwidth at 2.5 GHz center frequency and (**b**) quadruple the signal with 500 MHz bandwidth; (**c**) IF signal with 500 MHz bandwidth at 2.5 GHz center frequency and (**d**) quadruple the signal with 2 GHz bandwidth.

The proposed radar transceiver with photonic elements has two ISAR image modes, and each mode consists of waveforms with different bandwidths to compare the resolution of ISAR images obtained from each bandwidth experimentally. Figure 6a presents the IF signal with 125 MHz at 2.5 GHz, and this signal is quadrupled to 500 MHz at 10 GHz through optical elements and radio frequency (RF) electronics as shown in Figure 6b. The wideband characteristics of the signal can be obtained by controlling the bias voltages of the DP-MZM and the output signal, such as optical power and RF signal. The optical transceiver module has been constructed to observe the output signal while adjusting the bias value in real time. Moreover, the temperature of optical components is a key factor to achieving the second-order modulation of DP-MZM; thus, the test port with thermoelectric cooling (TEC) was adopted for use in the optical transceiver module in order to continuously generate a stable signal.

To obtain broad bandwidth for high-resolution ISAR images, the IF signal of another ISAR mode was designed with 500 MHz at 2.5 GHz as shown in Figure 6c. Figure 6d represents the transmit signal with a bandwidth of 2 GHz at 10 GHz to identify features of targets clearly in the cases of flights close to the radar transceiver system [12].

**Figure 7:**
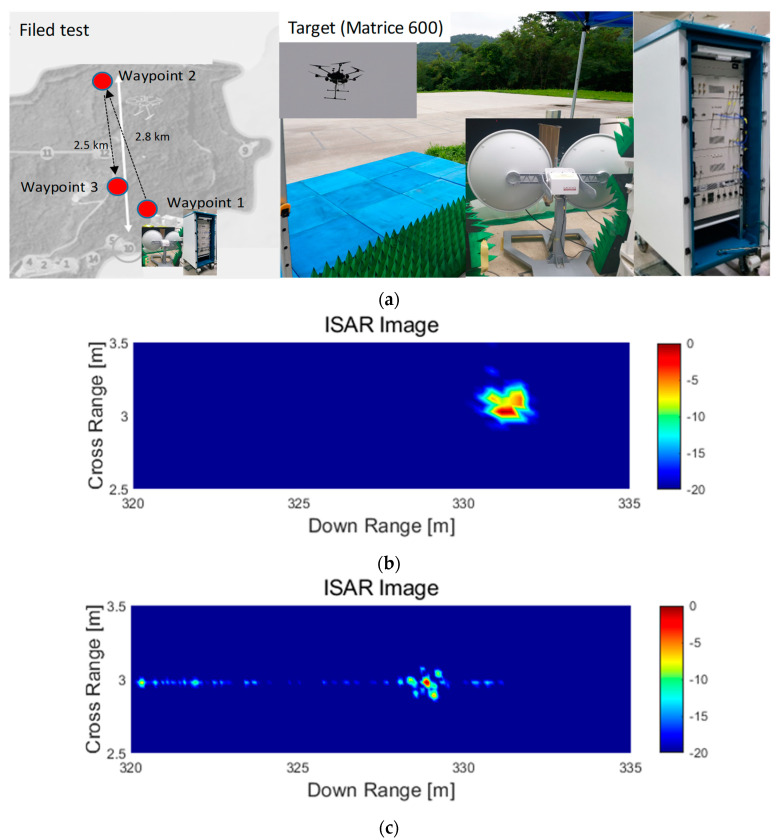
Obtained ISAR images of the drone target using the proposed FMCW radar with photonic elements having bandwidths of (**a**) the field test, (**b**) 500 MHz, and (**c**) 2 GHz.

Figure 7 shows ISAR images for the drone target at 330 m distance using the proposed FMCW radar with photonic elements according to different bandwidths. Figure 7a shows the scenario for the field test with the measurement environment; the target (Matrice 600) has waypoints with waypoint 2 being 2.8 km away from the radar site with an altitude of 500 m. Figure 7b,c present ISAR images when the bandwidths of the proposed radar system are 500 MHz and 2 GHz, respectively. The range resolution of the proposed system can be improved from 2 m to 0.5 m when the bandwidth of the proposed radar system increases. The results show that the proposed radar system is capable of acquiring high-resolution ISAR images, and it is confirmed that the body and motors in the wing part of the drone are more visible at 2 GHz than at 500 MHz.

## 4. Conclusions

This letter proposed a FMCW radar with photonic elements, and the signal generation with the use of photonic elements was performed to obtain wideband characteristics. It was shown by field tests that the proposed radar system is capable of detecting a small drone target at 2.5 km distance, as expected through the budget analysis. In addition, ISAR images of a small drone target with RCS of 0.1 m^2^ at 0.33 km distance were acquired. ISAR images were obtained by changing the bandwidth from 500 MHz to 2 GHz, and the ability of acquiring high-resolution ISAR images was observed in order to verify the effectiveness of achieving wideband characteristics of the proposed radar system. The results demonstrated that the proposed transceiver is suitable for the radar system to offer high-resolution ISAR images that can detect desired targets.

## Figures and Tables

**Table 1:** Parameters of the proposed radar transceiver.

Parameters	Quantity	Values
*P_t_*	Transmit power	20 W
*G_t_*	Transmit antenna gain	37 dBi
*G_r_*	Receive antenna gain	37 dBi
*G_p_*	Signal processing gain	21 dB
*F*	Noise figure	15.57 dB
*L_T_*	Total loss	13.58 dB
*K*	Boltzmann’s constant	1.38 × 10^−23^ J/K
*T*	Standard temperature	290 K
min(*S*/*R*)	Minimum SNR	13 dB

## Data Availability

Not applicable.
